# The Will to Destroy: Mrs. Fenwick's and the Central Committee's Statement Examined

**Published:** 1916-12-09

**Authors:** 


### THE MATRONS' AND SISTERS' DEPARTMENT

### THE WILL TO DESTROY.

### Mrs. Fenwick's and the Central Committee's Statement Examined.

Having regard to the importance of enabling our readers to form a sound judgment as to the grounds of difference between the Central Committee for the State Registration of Nurses and the College of Nursing, we are publishing this week the statement "lately issued under the authority of the Central Committee for the State Registration of Nurses." The necessity on the part of the Central Committee of publishing some such statement as an apology for what Mr. Stanley has described as an "abrupt termination to negotiations for an agreed Bill at a moment when substantial progress was believed to have been made" is obvious in view of the protests against the Committee's action already raised by the Officers of the Royal British Nurses' Association, and by the very important Association for the Promotion of the Registration of Nurses in Scotland.

#### Mr. Stanley Justified.

The first part of the statement is of an historical character, which the *British Medical Journal* omits, but it includes the best justification for the new departure identified with Mr. Stanley's name by the admission that the cause of State Registration, "which has been before the British public for the past quarter of a century," had made no more substantial progress up to 1914 than the passing of the First Reading of the Central Committee's Bill, which First Reading, contrary to almost invariable practice, was not permitted without a division being taken. This fact demonstrates very clearly the strength of the opposition which would have had to be overcome at each of the later stages of the measure, and meant ultimate rejection.

#### The Movement in a Quagmire.

It was, indeed, high time to lift the movement out of the quagmire in which it was sinking, to attempt to bring about a change of methods and *personnel*, and for a barren scheme of Registration to substitute the establishment of a College of Nursing, having Registration as its first, but by no means its only, object, properly based upon the voluntary co-operation of the leading nurse-training schools throughout the country, which had hitherto been ignored by the Central Committee. The names of those whose services Mr. Stanley has been enabled to enlist upon the Council of the College indicate very clearly how completely his aspirations for such a scheme have been accomplished.

#### Allegations to be Safely Disregarded.

It is difficult to understand upon what grounds the College is accused of "a breach of agreement" at a time when it is admitted that no agreement had been concluded. It would be equally reasonable to accuse the Central Committee of bad faith because at a certain stage of the negotiations they saw fit to break them off. Such allegations would seem to be made merely, as the lawyers say, to "import prejudice," and may safely be disregarded. As well might it be alleged that the Central Committee had made a breach of agreement because for the first time in the statement now published it is urged as "a fundamental objection in the Bill of the College" that a "voluntary College is to be associated with a statutory Council." Such an association was assumed from the very first, and the Fourth Draft of the Bill, Section 2 (2), reads: "The College of Nursing shall for the purpose of this Act act by the Council thereof, which shall be entitled 'The General Nursing Council,' and is hereinafter called 'The Council.'" *Against this arrangement no adverse criticism was ever directed by the Central Committee, yet those responsible for the statement have now seen fit to declare that this difficulty is, after all, fundamental.*

#### Mere Trifling with the Medical and Nursing Professions.

No amount of verbiage can obscure the plain fact that for some reason not perhaps difficult to divine the Central Committee has determined not to table the names of its representatives on the Provisional Council. All talk about the direct representation of nurses is but the proverbial red herring. Neither in the College Bill nor in the Committee's Bill has the nursing profession *direct* representation on the first Council. From the nature of the case no direct representation is, indeed, possible until the electing body has been constituted, and the electorate is the body of nurses who form the Register. The College is already compiling such a Register, and thus paving the way at once for direct representation. The Central Committee's Bill does and can do nothing in this direction. It waits—oh, how patiently!—until the Registration Bill has been passed by Parliament, and then only is the work of actual registration to be taken in hand; and only when the President of the Council is satisfied that enough nurses have been registered will there be, some day, an election of direct representatives on the permanent Council of Nursing. Surely all this is but trifling with the intelligence of the medical and nursing professions.

#### The Real Difference and Some Flaws.

The real difference between the College Bill and the Committee's Bill can hardly be more plainly put than it is in Mr. Stanley's letter (see The Hospital, November 18, p. 145, col. 1. clause v. (1)).

The Central Committee's Bill excludes names, and suggests a list of appointing bodies; the Council's Bill proposes to set out the actual names of the members of the First General Nursing Council. It is a question which method is more likely to conciliate opposition and to facilitate the passing of the Bill.

The Central Committee's statement makes no effort to grapple with Mr. Stanley's criticism of the flaws in their Bill—viz., that there is no representation of Poor-Law Authorities or Poor-Law nurses; no representation of Scotland or Ireland; and no escape from the difficulty of choosing six out of the whole of the nurse-training schools of the country without causing jealousy amongst the excluded. Can it be seriously contended that any essential difference is disclosed in "the statement" to justify the Central Committee in breaking off negotiations, particularly when Mr. Stanley repeats that he has no intention of receding from the position originally taken up by him that the Central Committee should have as many nominees on the Provisional Council as the College?

Points of Secondary Importance.

Admitted that agreement as to the *personnel* of the First Council had been reached, the exact proportional composition of the permanent Council becomes of secondary importance, always provided that, as stipulated in the College Bill, but not in the Central Committee's Bill of 1914, two-thirds of its members are to be elected by the nurses on the Register. Given the acceptance of this proviso, the rest might have been left to the First Council, which has to frame rules for the appointment of the permanent Council, rules which do not come into force without the sanction of the Privy Council, which can be opposed by interested parties before the Privy Council, and must in any case be laid before Parliament. The College Bill has been lightened of all that is not essential, and thus will its passage be rendered easier. Nor can it be doubted that Parliament will be prepared to concede to the College a large measure of responsibility and authority as a known body chosen from amongst persons of light and leading in the Government, in the medical profession, in the nurse-training schools, and from amongst nurses in the actual practice of their profession. The other two points to which the Central Committee attach importance—viz., the registration of existing nurses, and the definite curriculum required to qualify for registration, have been so conclusively dealt with in Mr. Stanley's letter (see The Hospital, November 18, pp. 145 and 146) that it is unnecessary to do more than refer our readers to what he has said.

Declared Reasons for the Break Entirely Inadequate.

For the rest, we have confined ourselves here to a brief examination of the statement issued by the Central Committee, and have shown how entirely inadequate are the reasons alleged for breaking off negotiations with the College for an agreed Bill. Later on we may have something to say of the unscrupulous way in which endeavours are being made in certain quarters to prejudice nurses against the College and the policy for which it stands. Whatever else may be said, there can be no two opinions that the Central Committee's reasons, as set forth in their "statement" (p. 207) demonstrate to conviction that there were, in fact, no adequate grounds for breaking with the College.

